# The Advanced Glycation End-Products (AGE)–Receptor for AGE System (RAGE): An Inflammatory Pathway Linking Obesity and Cardiovascular Diseases

**DOI:** 10.3390/ijms26083707

**Published:** 2025-04-14

**Authors:** Elena Vianello, Antonio P. Beltrami, Aneta Aleksova, Milijana Janjusevic, Alessandra L. Fluca, Massimiliano M. Corsi Romanelli, Lucia La Sala, Elena Dozio

**Affiliations:** 1 Department of Biomedical Sciences for Health, Università degli Studi di Milano, 20133 Milan, Italy; elena.vianello@unimi.it (E.V.); mmcorsi@unimi.it (M.M.C.R.); lucia.lasala@unimi.it (L.L.S.); 2 Experimental Laboratory for Research on Organ Damage Biomarkers, IRCCS Istituto Auxologico Italiano, 20149 Milan, Italy; 3 Department of Medicine, Università degli Studi di Udine, 33100 Udine, Italy; antonio.beltrami@uniud.it; 4 Azienda Sanitaria Universitaria Friuli Centrale, 33100 Udine, Italy; 5 Department of Medical Surgical and Health Sciences, Università degli Studi di Trieste, 34129 Trieste, Italy; aaleksova@units.it (A.A.); mjanjusevic@units.it (M.J.); alessandralucia.fluca@units.it (A.L.F.); 6 Cardiothoracovascular Department, Azienda Sanitaria Universitaria Giuliano Isontina, 34100 Trieste, Italy; 7 Department of Clinical and Experimental Pathology, IRCCS Istituto Auxologico Italiano, 20149 Milan, Italy; 8 IRCCS Multimedica, 20138 Milan, Italy

**Keywords:** advanced glycation end-products, atherosclerosis, apoptosis, adipose tissue, cardiovascular diseases, cardiovascular risk, heart failure, inflammation, obesity, oxidative stress, receptor for advanced glycation end-products

## Abstract

The AGE (advanced glycation end-products)–RAGE (receptor for AGE) system is a pro-inflammatory pathway that contributes to the pathogenesis of obesity and obesity-related cardiovascular disorders (CVD). Circulating AGE and the soluble form of RAGE (sRAGE) has been suggested as a potential biomarker of CVD related to obesity. In this study, we aim to (1) summarize the current knowledge about the role of obesity in the onset and progression of CVD, (2) discuss the role of the AGE–RAGE system as a pathway promoting obesity and linking obesity to CVD, and (3) highlight available strategies for reducing AGE–RAGE system activation and the associated beneficial effects.

## 1. Introduction

The onset and progression of cardiovascular diseases (CVD) can result from metabolic changes caused by weight gain and obesity, particularly visceral obesity. In contrast to subcutaneous adipose tissue (SAT) depots, in which adipogenesis is the main mechanism promoting fat accumulation, visceral fat (VAT) increase is mainly mediated by a hypertrophic response. However, due to the reduced plasticity, hypertrophic visceral adipocytes undergo apoptosis, release lipids outside and trigger an immunological response. Lipids, lipid-derived metabolites, adipokines, and cytokines released by infiltrating immune cells promote a chronic inflammatory response that impairs adipose tissue metabolism and has detrimental effects on many organs, including the cardiovascular system [1,2,3].

Previous research has shown that the AGE (advanced glycation end-products)–RAGE (receptor for AGE) pathway promotes adipose tissue expansion, inflammation, atherogenesis, and disrupts cardiomyocyte homeostasis [4,5,6]. AGE and the soluble form of RAGE (sRAGE) are potential biomarkers of obesity-related CVD. Blocking this pathway appears to have promising therapeutic potential. The aim of this review is to address the role of AGE, RAGE, and sRAGE as pathogenetic, diagnostic, and prognostic molecules in obesity and obesity-related CVD, as well as to describe available strategies for reducing AGE–RAGE system activation and the associated beneficial effects.

## 2. Obesity and Cardiovascular Risk

### 2.1. Adipose Tissue Expansion in Obesity

Adipose tissue is a multifunctional organ that, by producing and secreting many different molecules, can affect the homeostasis of other tissues, including the cardiovascular system. However, VAT and SAT can differently contribute to CVD risk [7]. VAT expansion occurs in response to over-nutrition and involves adipocyte hypertrophy. Both the clearance of circulating lipids and de novo lipogenesis increase mature adipocyte diameter from <20 to 300 μm [8]. Unlike SAT, preadipocyte differentiation plays a smaller role in VAT expansion than hypertrophy. This suggests that VAT has fewer adipocyte progenitors and/or less activity than SAT. Interestingly, adipocyte hypertrophy and hyperplasia are inversely correlated: the smaller the adipocytes, the higher the adipogenic rate [9]. When the maximal adipocyte volume is reached, cell death increases and structures that resemble crowns, mostly composed by M1-polarized macrophages, appear around dead cells [10]. The local environment becomes, therefore, enriched of dying adipocytes and inflammatory cells, which can play different functions, including the clearance of dead adipocytes, enhancement of inflammation, lipid scavenging, extracellular matrix remodeling, and angiogenesis [11,12,13,14].

Inflammatory cells are activated in response to chemokines like monocyte chemotactic protein-1 (MCP-1), which is generated by hypertrophic adipocytes. A small proportion of macrophages are generated by the modulation of resident cells, whereas most of them derive from circulating monocytes [15,16]. In healthy adipose tissue, resident macrophages, also known as M2 macrophages, express markers such as arginase-1, macrophage galactose type C-type Lectin, and mannose receptor and promote adipose tissue homeostasis by producing anti-inflammatory mediators such as interleukin-10 [17,18,19]. The transition to pro-inflammatory M1 cells is supported by a “metabolic activation” that primarily involves molecules such as palmitate, glucose, and insulin, and it is different from the one triggered during infection. In fact, metabolically activated macrophages exhibit a distinct set of cell surface markers, including ATP Binding Cassette Subfamily A Member 1, cluster of differentiation 36, and perilipin 2. Tumor necrosis factor α (TNFα) and interleukin 1 β are the major pro-inflammatory cytokines released by these cells [20].

Adipose tissue hypoxia, which develops when the mass expands, can further activate the inflammatory response and contribute to the generation of oxygen free radicals, reactive oxygen species (ROS), and oxidative stress. Hypoxia also induces insulin resistance (IR), inhibits preadipocyte differentiation, and promote fibrosis [21]. The alteration of extracellular matrix remodeling, which includes the deposition of collagen fibers both around adipocytes and dispersed throughout the tissue, and the reduction in the number of brown/beige cells are additional events associated with mass expansion. As a result, a dysfunctional tissue with decreased thermogenesis and plasticity is produced [9,22]. Beige adipocytes reside within white adipose tissue. While white adipocytes store energy, beige adipocytes burn it and protect against obesity and related metabolic disorders, such as hyperglycemia and hyperlipidemia. In obesity, increased production of inflammatory mediators in adipose tissue limits beige adipogenesis, promotes brown-to-white plasticity, reduces browning extent, and impairs thermogenic activities. These pathways further promote adipose tissue growth and metabolic alteration [23]. Molecules released from adipose tissue, such as free fatty acids (FFA), inflammatory cytokines, lipid peroxidation products, AGE, and carbonylated proteins, can all contribute to explaining the relationship between dysfunctional adipose tissue and CVD risk. Increased circulating FFA can be attributed to the increased FFA efflux from adipose tissue, increased lipolysis due to reduced insulin sensitivity, and increased postprandial lipid spillover [24,25,26]. Inadequate angiogenesis in obesogenic adipose tissue can also disrupt adipogenesis and promote metabolic disorders [27].

### 2.2. How Can Obesity Affect the Heart and the Vascular System?

The etiopathogenesis of CVD is multifactorial. The different factors that contribute to CVD risk may be classified into non-modifiable and modifiable. The first category comprises age, race/ethnicity, and gender. Dietary habits, physical inactivity, obesity, diabetes mellitus (DM), dyslipidemia, hypertension, and smoking are all controllable factors which can often coexist. Excellent previous publications detailed how these factors can raise the risk of CVD [28,29,30,31]. Here, we will briefly discuss how obesity, mainly visceral obesity, can contribute to onset and progression of CVD.

Regarding the heart, it is known that about 20–30% of the energy needed by a healthy adult heart comes from glucose and 60–80% comes from β-oxidation. In the fed state, the heart can respond to insulin by switching from FFA to glucose metabolism; during fasting, it can respond to high-plasma FFA by switching back to FFA oxidation [32]. Nonetheless, obesity alters cardiac FFA acid metabolism by affecting uptake and oxidation processes. In the heart, FFA absorption and β-oxidation are crucially regulated by the supply of FFA. Enhanced cardiac expression of transporters like CD36, which is upregulated by hyper-insulinemia, can enhance FFA uptake in obesity [33]. Increased supply and absorption of FFA and impaired fatty acid β-oxidation can contribute to heart lipid accumulation. However, in animal and human models of obesity, both elevated and lowered cardiac β-oxidation rates have been reported. Since energy metabolism in obese hearts is primarily dependent on endogenous lipid store, the lack of measurement of endogenous FFA β-oxidation rate—which has been demonstrated to be at least as high as that of plasma FFA [34,35]—could be a plausible explanation of the different results across studies. According to several research, β-oxidation appears to increase before the development of cardiac dysfunction and decline when the heart starts showing the signs of lipid accumulation and contractile failure [36,37,38,39,40]. When the amount of available endogenous and exogenous FFA exceeds the capacity of β-oxidation, different lipids, including triglycerides, diacylglycerols, ceramides, long-chain acyl CoAs, and acylcarnitines, start to accumulate and have detrimental effects on cardiac cells [32]. Even if ceramide accumulation in obesity has been considered an adaptive response to increased FFA influx, ceramides can induce cardiac lipotoxicity. In fact, they can affect cell membrane fluidity and dynamics, promote inflammation through the activation of the NLR family pyrin domain containing 3 inflammasome, and induce apoptosis [41,42]. In obesity, the accumulation of ceramides in cardiomyocytes also appears to play a crucial role in cell death and CVD pathophysiology, including atherosclerosis and heart failure [43].

Different outstanding publications have also addressed the role of inflammation, lipid overload, lipid peroxidation, and the synthesis of other toxic chemicals in promoting the dysfunction of endothelial cells, arterial smooth muscle cells, and macrophages in the vessels [44,45]. Furthermore, by targeting the liver, and through changes in liver-derived lipoproteins, clotting factors, and inflammatory mediators, obesity-derived mediators can also promote atherogenesis [46]. Clinical and epidemiological research demonstrates a high correlation between inflammatory markers and the risk of CVD. The most accessible and reliable inflammatory marker for clinical use is high-sensitivity C-reactive protein. Its role as a biomarker has been well established. Some mechanistic investigations also suggested its potential involvement in the pathogenesis of atherosclerosis [47].

Multiple variables (e.g., inflammation, IR, metabolic alterations) influence the relationship between obesity and CVD, and all of the modifiable factors mentioned above are possible targets for lowering the risk. Strategies aimed at reducing FFA flow, ceramide synthesis, and the harmful effects of inflammatory mediators and pro-oxidant compounds might have cardio-protective benefits. However, if we assume that obesity-related CVD is driven by unhealthy VAT expansion, identifying specific targets to minimize VAT expansion and optimize its metabolism remains a challenge in the reduction in the overall CVD risk.

In the next sections, we will discuss how obesity-derived AGE and RAGE activation can affect heart and vessel homeostasis, thus representing a link between obesity and CVD risk.

## 3. Advanced Glycation End-Products (AGE): From Synthesis to Mechanisms of Action

### 3.1. AGE Synthesis and Classification

AGE are a heterogeneous set of chemicals formed by the non-enzymatic reaction between the carbonyl groups of reducing sugars, such as glucose, fructose, or their metabolites, and the amino groups found in proteins, lipids, and nucleic acids. Lysine and arginine residues of proteins are the main targets involved in the synthesis of AGE. Many distinct pathways can be involved in the synthesis of AGE, and some of them are tightly interconnected. A detailed description of the chemical pathways leading to AGE can be found in previous publications from Chen [48]. Increased glucose concentration, oxidative stress, and inflammation can all enhance AGE buildup by producing highly reactive carbonyl intermediates and interacting with other bio-active molecules. As a result, increased levels of AGE can be detected in chronic diseases characterized by high levels of glucose, inflammatory mediators, and ROS, such as DM, CVD, obesity, and chronic kidney disease (CKD) [49,50]. To be noted, in CKD, AGE also accumulates due to impaired renal filtration and detoxification [51].

AGE can be classified based on the source (endogenous or exogenous), precursor molecules [glucose, fructose, glycolaldehyde, glyceraldehyde, methylglyoxal (MGO), glyoxal (GO), 3-Deoxyglucosone], chemical structures (linked or non-cross-linked), ability to emit fluorescent light, molecular weight (12 kDa is the cut off for classification into low or high molecular weight), amino acids involved in their synthesis (lysine, arginine, cysteine), and toxicity [52]. According to the hypothesis of Takeuchi M., toxic AGE (TAGE) are a category of AGE made up of glyceraldehyde and intracellular proteins, and they are regarded as the most dangerous [53]. The increased availability of sugars in the modern diet promotes intracellular synthesis of TAGE by increasing glyceraldehyde production and subsequent interaction with intracellular proteins. TAGE may accumulate within cells, and they can induce cell dysfunctions leading to cell necrosis and the further release of TAGE into the bloodstream. TAGE can then cause oxidative stress and inflammation also in cells other than those that produced by interacting with RAGE [53]. AGE produced by metabolic processes different from glyceraldehyde metabolism are called nontoxic and they are considered products of “avoiding pathways” that trap reactive dycarbonyl molecules and limit TAGE production. The intracellular toxic effects of TAGE have been reported in various cell types, including cardiomyocytes, cardiac fibroblasts, hepatocytes, pancreatic β-cells, neurons, and myoblasts [54,55,56,57,58,59]. Only a few TAGE structures have been revealed so far. Proteomic investigations revealed four distinct glyceraldehyde-derived AGE structures [55]. Blocking these TAGE, but no other AGE, has been shown to minimize some harmful events in vitro. However, some other studies revealed that even AGE designated as non-toxic, such as carboxymethyllysine (CML), may induce damage, particularly in the cardiovascular system [60,61,62]. These data emphasize the importance of carrying out further investigations to uncover the structures of unknown AGE and better define the roles of the different AGE as pathogenetic agents and/or disease biomarkers.

### 3.2. AGE Accumulation

AGE can be quantified using different assays, including fluorimetric methods, high-performance liquid chromatography, gas chromatography coupled with mass spectrometry, liquid chromatography–tandem mass spectrometry, and enzyme-linked immunosorbent assay (ELISA). Each of these approaches has various advantages and disadvantages, which may include cost, sensibility, the complexity of the procedure, and the type of AGE that can be quantified. Ex vivo AGE quantification can be performed using biological fluids as well as tissues. In vivo, skin autofluorescence can be measured. Because AGE accumulates in tissues, the skin is an easily accessible location for this purpose. The limitations of both in vitro and in vivo fluorescent methods are the inability to detect non-fluorescent chemicals and the expression of data as relative units of measurement [63].

Plasma AGE concentration reflects the amount of AGE introduced by food, the amount of AGE produced by cells, the modification of AGE-containing extracellular matrix proteins, and the amount of AGE detoxified by the liver and kidneys. The liver can convert AGE into lower-molecular-weight molecules. Cells such as macrophages, Kupffer cells, and endothelial cells can internalize AGE via the scavenger receptor for AGE (AGE-R1) and convert them into low-molecular-weight and soluble chemicals that can be further eliminated by the kidney [64,65]. Plasma AGE can therefore rise because of increased intake and endogenous production, as well as reduced renal filtration [51]. Other endogenous defense strategies against AGE include AGE-R2 and AGE-R3, as well as the macrophage scavenger receptors ScR-II and CD-36 [66]. All these molecules are clearance receptors that remove AGE from the circulation, accelerate their breakdown, and inhibit AGE-damaging effects [64]. AGE-R1 was the first identified; its level rises with increasing AGE, but it is downregulated by consistently high AGE levels [67]. AGE-R2 lacks binding capabilities; however, by interacting with AGE-R3, it promotes the stability of the receptor complex [68,69]. Interestingly, prolonged exposure to AGE enhances AGE-R3 expression [70]. Several enzymes, such as aldose reductase, aldehyde dehydrogenase, and glyoxalase-1 and -2 (GLO-1 and GLO-2) may regulate AGE synthesis. GLO-1 and GLO-2 break down dicarbonyl molecules, hence inhibiting AGE development. GLO-1 is the rate-limiting enzyme whose activity is significantly dependent on glutathione levels. Oxidative stress can therefore increase AGE formation by depleting glutathione and decreasing GLO-1 activity [71].

### 3.3. RAGE-Independent and RAGE-Mediated Effects

AGE effects can be divided into two main categories: receptor-independent and receptor-dependent effects. The first group comprises the effects associated with the molecular alterations of proteins involved in AGE synthesis. These effects include changes in the molecular structure and biological characteristics of extracellular and intracellular proteins, alteration of receptor binding properties and signal transduction, impaired molecular clearance, loss of signaling between cells and the cellular matrix, loss of the mechanical characteristics of matrix proteins, and intracellular accumulation leading to cell dysfunction [56,72,73,74,75,76,77].

The receptor-dependent effects are mediated by RAGE. RAGE is a type 1 transmembrane receptor belonging to the immunoglobulin superfamily. It consists of an intracytoplasmic tail, a transmembrane region, and an extracellular portion divided into two constant domains (C1 and C2) and a variable domain (V). The latter is responsible for binding extracellular molecules, whereas the C domains play critical roles in stabilizing V-domain interaction with ligands and are involved in receptor homodimerization. Besides AGE, several other molecules can bind to RAGE and activate RAGE-mediated responses, including S100 proteins, lipopolysaccharide, β-amyloid peptide, and high-mobility group box 1 (HMGB1) [78]. In humans, the RAGE gene extends for 3080 bp and it is located on chromosome 6, within the human leukocyte antigen class III, near its junction with class II. In physiological conditions, RAGE is expressed at low levels in many organs by several cell types, including endothelial cells, monocytes/macrophages, hepatocytes, T lymphocytes, neuronal cells, glomerular epithelial cells, and vascular smooth muscle cells. Once triggered, it stimulates inflammatory pathways that lead to tissue regeneration and repair. On the other hand, if the blood levels of RAGE ligands remain high, RAGE activation boosts its own expression, thus enhancing its downstream effects [79,80]. Once activated, RAGE can activate several intracellular pathways that can be different according to cell type and tissues These pathways include Janus Kinase 2/Signal Transducer and Activator of Transcription protein 3, increased expression of ROS, Mitogen-Activated Protein Kinases (MAPK) p38, and Extracellular Signal-Regulated Kinase 1/2, and nuclear translocation of nuclear factor kappa-light-chain-enhancer of activated B cells. The final effect is the modulation of gene expression. The most affected genes are those involved in the pro-inflammatory response, acquisition of migratory capabilities, activation of apoptotic machinery, and regulation of cell proliferation [81].

Besides the full-length RAGE, additional isoforms were discovered at the mRNA and protein levels. These isoforms are characterized by mRNA transcripts that contain missing or additional exons/introns, or portions of them, due to alternative splicing of the RAGE pre-mRNA. Numerous spliced forms have been identified so far and classified according to the Human Gene Nomenclature Committee, with the first variant called RAGE_v1 [82]. Notably, RAGE_v1, which is due to inclusion of part of intron 9 and deletion of exon 10, produced a secretory form (esRAGE–endogenous secretory RAGE) that can be detected in biological fluids [82]. Two main mechanisms are involved in the synthesis of soluble RAGE (sRAGE): (1) alternative splicing and (2) shedding of the full-length membrane receptor (cRAGE) [83,84,85]. Despite the fact that these molecules are produced by different mechanisms, both cRAGE and esRAGE work as decoy receptors. By binding to AGE in the circulation, they prevent RAGE activation and RAGE-induced effects. cRAGE is the main circulating form in healthy conditions. esRAGE levels are typically decreased at increasing membrane RAGE expression. This mechanism seems to favor the harmful pathway over the protective one. IR, hypoxia, and inflammation can all modulate the enzymes that can cleave RAGE [86,87].

The reduced sRAGE levels observed in obesity, IR, metabolic syndrome, and CVD could be due to the upregulation of membrane RAGE expression, the reduced RAGE cleavage, and the downregulation of esRAGE expression and secretion [88,89,90,91]. In contrast, the enhanced cleavage of membrane RAGE might explain the up regulation of sRAGE level found in some acute cardiovascular illnesses, DM, and CKD. In this scenario, the cleavage could be regarded a protective event: decreasing membrane RAGE can attenuate the impact of its activation, while increasing sRAGE can prevent circulating RAGE ligands [92,93,94]. This suggests that sRAGE and its forms can play different roles as disease biomarkers according to the clinical setting.

### 3.4. sRAGE as a Biomarker

sRAGE and its variants can be measured in several biological fluids. One of the easiest methods is ELISA. ELISA allows for the quantification of total sRAGE and esRAGE. Total sRAGE can be identified using a primary antibody that detects an extracellular region of the receptor that is common to all forms, and the second can be quantified using a primary antibody that recognizes an intracellular region that is lacking in cRAGE. cRAGE can be then calculated by subtracting esRAGE from total sRAGE. Despite numerous studies indicating its potential role as a biomarker, sRAGE has not yet been used in the clinical practice. In healthy condition, sRAGE mean levels do not differ between men and women, but are lower in people over 50 years [95].

The pathogenic role of the AGE–RAGE axis has been described in many organs including the pancreas, adipose tissue, heart, vessels, lungs, kidneys, gut, bones, and muscles. The activation of this pathway, along with the synthesis of AGE on specific proteins, can lead to organ dysfunction by promoting β-cell loss, IR, matrix protein crosslinking and impaired turn-over, fibrosis, inflammation, reduced endothelial nitric oxide synthase 3 expression, increased pulse pressure, podocyte apoptosis, increased gut permeability and altered microbe composition, reduced osteoblast and increased osteoclast maturation, promoted muscle satellite cell dysfunction, platelet hyperreactivity, and adipose tissue growth [96,97,98,99,100,101,102,103,104,105]. In the next sections, we will describe in detail the role of the AGE–RAGE system in the pathogenesis of obesity.

## 4. Adipose Tissue: Source and Target of AGE

### 4.1. AGE Accumulation in Adipose Tissue

AGE can accumulate in adipose tissue because they are locally produced due to the increased glucose availability and/or oxidative stress, or because they are supplied by the circulation. Locally produced AGE can be released into circulation and, by binding to RAGE, they can exert their effects in tissues other than those on which they are synthesized, such as in the cardiovascular system. Most of the studies on obesity focused on plasma AGE quantification. In a recent publication, Şermin Durak et al. quantified some AGE forms (GO, malondialdehyde and MGO) in the adipose tissue, liver, and kidneys of mice fed a high-fat diet [106]. Contrary to expectations, GO and MGO levels were found to be lower in the adipose tissue of obese mice than controls. This reduced accumulation was mainly attributed to the increased inflammation and oxidative stress, which reduce the levels of these compounds by promoting their interactions with other proteins. However, these results agreed with previous observations suggesting lower AGE levels in adipose tissue than in other organs [107] due to increased metabolism and greater leakage. Differently, Katrien H.J. Gaens et al. observed an increased accumulation of CML in obese human SAT and VAT, with significant differences between the two depots only in severe obese individuals (BMI, body mass index, >40 kg/m^2^). CML-modified proteins were particularly evident in adipocytes, CD68-positive macrophages, and CD31-positive endothelial cells. Also RAGE was upregulated and co-localized with CML [108]. By comparing these studies, we may conclude that accumulation is primarily determined by the chemical structure of the AGE being investigated. Other forms of AGE and other RAGE ligands, including AGE-albumin and S100b, were measured in VAT and SAT of aging rats on a regular chow diet [109]. Macrophage infiltration, AGE-albumin and S100b accumulation, and RAGE expression were found to be higher in VAT of 28-week-old rats compared to 3-week-old rats, but equal on SAT. As a result, the AGE–RAGE system appears to contribute also to the pathogenesis of aging-related VAT disease.

### 4.2. AGE Effects in the Adipose Tissue

Different studies highlighted the role of RAGE in adipose tissue growth, inflammation, and IR. Ueno H et al. [4] evaluated body weight, the weight of epididymal adipose tissue, and epididymal adipocyte size between apoE−/−RAGE+/+ and apoE−/−RAGE−/− mice fed a standard chow diet or an atherogenic diet. They found that RAGE deletion decreased body weight, without affecting food intake, and reduced epididymal adipose tissue weight and epididymal adipocyte size. Serum adiponectin, an anti-inflammatory cytokine, was strongly and inversely associated with epididymal fat weight and adipocyte size. It was also significantly greater in apoE−/−RAGE−/− mice than apoE−/−RAGE+/+ animals. The molecular processes underlying RAGE control of adiposity and the cell types that express RAGE were not explored, and, up until now, they are not fully understood. RAGE activation in inflammatory cells cannot be ruled out, even though the authors barely detected crown-like structures in the epididymal fat of both groups. Monden M. et al. [5] also found a correlation between RAGE, adipocyte hypertrophy, and insulin sensitivity. They observed that the overexpression of RAGE in 3T3-L1 cells via adenoviral gene transfer was linked with adipocyte hypertrophy, inhibition of glucose transporter type 4 (GLUT4) and adiponectin mRNA expression, and decreased insulin-stimulated signaling and glucose uptake. Small interfering RNA inhibited RAGE, which greatly reduced adipocyte hypertrophy. Furthermore, RAGE−/− animals had considerably lower body weight, epididymal fat weight, epididymal adipocyte size, increased blood adiponectin, and improved insulin sensitivity. RAGE-mediated modulation of toll-like receptor (TLR) 2 was identified as one of the pathways linking RAGE to obesity and insulin sensitivity. Rodents fed high-fat diets developed systemic inflammation and obesity and displayed increased expression of the RAGE ligand HMGB1 and CML-AGE epitopes in adipose tissue. RAGE genetic deficit lowered obesity and adipocyte hypertrophy. Furthermore, RAGE hematopoietic deficiency or sRAGE therapy provided partial protection against weight gain and inflammation [110]. The role of RAGE in obesity-induced inflammation was also confirmed by Du et al. [111]. RAGE deficiency protected against obesity-induced inflammation and IR in a sex-dependent manner. Female RAGE-deficient mice had better glucose and insulin tolerance, a lower expression of pro-inflammatory genes in M1 macrophage, and a higher expression of antioxidant genes [111].

In human preadipocytes, CML activated inflammation and adipogenesis through RAGE [108]. RAGE deletion in db−/− obese mice enhanced inflammatory profile and glucose homeostasis and decreased CML trapped in adipose tissue. RAGE-mediated CML accumulation in adipose tissue might explain why obese individuals have low plasma CML levels, adipokine dysregulation, and IR [108]. Unoki et al. demonstrated that glucose-, glyceraldehyde-, or glycolalde-derived AGE inhibited the differentiation of 3T3-L1 pre-adipocytes, promoted the generation of ROS and MCP-1, and reduced glucose uptake in the absence or presence of insulin through RAGE activation [112]. GO-AGE inhibited insulin-stimulated glucose uptake in 3T3-L1 cells in a biphasic manner. Initially, glucose uptake increased by 168% within the first 8 h of incubation, but significantly decreased after 24–48 h. GO-AGE also enhanced lipid droplet production, and treatment with an anti-RAGE antibody inhibited this effect. The study found that RAGE-mediated early lipid synthesis, ROS generation, and TNFα production promoted IR [113]. AGE could also induce extracellular matrix remodeling in adipose tissue, regardless of RAGE activation. AGE stiffened the cell niche and modified the rheological viscoelastic properties of cultured cells, thus affecting cell signaling. Alterations in plasma cell dynamics resulted in decreased adipogenesis and impaired signal transduction, which can alter insulin signaling [114].

Sustained adipose tissue inflammation is mainly due to inflammatory mediators produced mostly by infiltrating macrophages. Regardless, we have to consider that the early phase of inflammation in adipose tissue is sustained by adipocytes, which can acquire pro-inflammatory properties. Through the secretion of interleukin-6, TNFα, and the alarmin HMGB1, they sustain an autocrine positive feedback loop and promote immune cells activation [112,115]. To be noted, in this phase, HMGB1 has been shown to exert its effects through RAGE, but not TLRs. Therefore, RAGE is supposed to be engaged early in the initiation and progression of adipose tissue inflammation and expansion.

A previous study from our group also suggested that RAGE may promote epicardial adipose tissue (EAT) dysfunction by increasing adiposity and impairing insulin signaling. Increasing RAGE expression resulted in a rise in EAT thickness, HMGB1, TLR4, and Myeloid differentiation primary response 88 levels, while decreasing GLUT4, adiponectin, and GLO1. We found significant relationships between RAGE and EAT thickness, as well as between RAGE and genes involved in inflammatory pathways and IR [116].

RAGE was also shown to regulate weight gain and adiposity by affecting energy expenditure and the browning process. This pathway leads to the appearance of beige adipocytes in white adipose tissue. By dissipating energy as heat, these beige adipocytes improve energy balance in adipose tissue. Mice with global or adipocyte-specific deletions of RAGE improved their metabolic recovery following fasting, cold challenge, or high-fat feeding [110,117,118]. Results obtained in RAGE−/− murine adipocytes indicated that RAGE inhibited thermogenesis [117]. RAGE also promoted lipid accumulation by inhibiting the β3-adrenergic receptor-mediated lipolytic pathway. Overexpression of RAGE in wild-type mice suppressed phosphorylation of hormone-sensitive lipase and p38 MAPK upon β-adrenergic stimulation, leading to the reduced expression/activity of uncoupling protein 1 and thermogenic programs [119]. These findings corroborate the role of RAGE as an immune modulator of energy expenditure.

**Figure 1:**
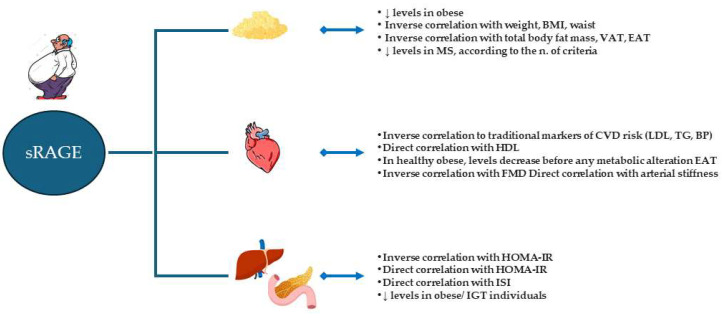
Effects of advanced glycation end-products (AGE)–receptor for AGE (RAGE) pathway on adipose tissue homeostasis. AGE can be produced locally, accumulate, cause local damage, and be released into the bloodstream by apoptotic cells. Adipose tissue may also be a target of AGE produced elsewhere. These AGE can primarily influence adipose tissue homeostasis by activating RAGE. Arrows mean the indicate increase or decrease concentrations. Some of the main pathways affected by RAGE activation are shown in the right box of the figure [89,91,120,121,122,123,124,125,126,127,128,129,130,131,132].

We can conclude that, in obesity, AGE and RAGE can regulate adipose tissue homeostasis at multiple levels, including adipocyte hypertrophy, matrix remodeling, inflammation, lipid accumulation, and IR. The mechanisms explaining these relationships have not been fully explored yet. As the activation of the AGE–RAGE system in the adipose tissue promotes dysfunctions, targeting this pathway might improve tissue homeostasis and reduce the detrimental effects of obesity on the cardiovascular system. The main effects associated with the AGE–RAGE pathway in the adipose tissue are summarized in Figure 1.

### 4.3. How Can the AGE and RAGE System Contribute to CVD?

The AGE–RAGE system can contribute to the burn of CVD in obesity by worsening adipose tissue metabolism, as previously stated, as well as by directly affecting the heart and vessels. The adipose tissue can be a direct source of some AGE, and it can contribute to the local production of these compounds by promoting metabolic alterations, i.e., diabetes, oxidative stress, inflammation, and IR, which accelerate AGE synthesis. The increased FFA uptake by the heart and the impairment of β-oxidation are other key mechanisms that increase the production of ROS and AGE synthesis [6,133,134]. A detailed discussion on how AGE and RAGE can accelerate the progression of numerous CVD by directly affecting cardiac and vascular tissues can be found in previous outstanding publications from our group [134,135,136,137,138,139,140].

Given that the AGE–RAGE system can promote cardiac and vascular remodeling, endothelial dysfunction, and inflammation, we have summarized the key molecular mechanisms underlying these effects. These data can explain how the AGE–RAGE system can contribute to the risk CVD in obesity, including heart failure, hypertension, and atherosclerosis.

The AGE–RAGE pathway can increase the production of extracellular matrix (ECM) in the cardiovascular system through profibrotic signaling [141]. This ECM buildup can occur from enhanced matrix protein production or reduced degradation, which disrupts communication between fibroblasts and ECM and encourages fibroblast differentiation into myofibroblasts. AGE can boost ECM accumulation by increasing the synthesis of pro-fibrotic factors, affecting cell–matrix interactions, and altering expressions of proteins related to oxidative stress and inflammation. Altogether, ECM accumulation and AGE-derived oxidative stress and inflammation can contribute to cardiac remodeling up to heart failure [141,142].

Endothelial cell (EC) permeability is essential for vascular health, and damage to cell membranes is common under stress. The process of repairing these membranes involves proteins that can fail, leading to tissue damage. Increased RAGE expression may hinder this repair process by reducing membrane flexibility [143]. Research has shown that RAGE negatively impacts membrane repair in human EC by affecting proteins like β-catenin. In addition to endothelial dysfunction, the AGE–RAGE axis can impair also the functions of smooth muscle cell and, by promoting the expression of matrix metalloproteinases, can induce extracellular matrix remodeling [144]. Clinical studies confirmed the association between the AGE–RAGE system and endothelial dysfunction, and sRAGE was described as a marker of hypertension [145,146].

In addition, atherosclerosis is associated with the AGE–RAGE system. Considering that the activation of this pathway promotes inflammation, endothelial damage, modifications in vascular smooth muscle cell function, and alterations in platelet activity [147], which are all key events in atherogenesis, it is not surprising that blocking this pathway could improve the size of atherosclerotic plaques [148].

## 5. AGE and sRAGE: Role as Early Biomarkers in Obesity and CVD Risk

AGE and sRAGE have been examined for their potential role as biomarkers in obesity and obesity-related CVD risk. Several research looked at the relationship between sRAGE and anthropometric measurements. Most of them reported a negative relationship between sRAGE levels, BMI, and waist circumference. A study conducted by our group on apparently healthy women with different BMIs found a negative correlation of sRAGE with overall fat mass and fat accumulated in specific depots, such as the VAT and the EAT [91]. A recent systematic review and meta-analysis reviewed the results of studies evaluating the relationship between sRAGE and obesity indices among adults [120]. This systematic review, which included 13 cross-sectional studies performed on healthy adults, adults with obesity, or obesity-related disorders, confirmed that individuals with obesity had significantly lower circulating sRAGE concentrations. Higher sRAGE levels were associated with lower BMI in 1865 apparently healthy individuals. Lower waist circumference (WC) was similarly associated with greater sRAGE concentrations in 1876 adults. Kathleen E. Davis and colleagues found that sRAGE was inversely correlated with weight, WC, and BMI in young individuals with BMI ranging from normal to overweight and obese [121]. The Northern Manhattan Study found that sRAGE levels decreased in the presence of metabolic syndrome, according to number of metabolic syndrome criteria, including central obesity [122]. sRAGE was strongly and inversely associated with the increasing number of metabolic syndrome components also in a group of male adolescents, in which BMI was an important predictor of sRAGE levels [123]. High circulating esRAGE levels, but not sRAGE, were linked to a reduced incidence of metabolic syndrome in Japanese adult men [89]. In obese adult women, sRAGE was lower than normal weight individuals and was inversely correlated with obesity and metabolic parameters [124]. Studies on children showed similar results. Obese children had lower plasma sRAGE levels and higher RAGE expression in peripheral blood mononuclear cells than overweight children (6–11 years old). sRAGE is associated negatively with RAGE and BMI. Multiple regression analysis identified RAGE level as an independent predictor of sRAGE [125]. This supports the idea that fat expansion affects the physiological mechanisms that regulate RAGE cleavage and protect against RAGE activation. Our recent observation about the existence of an independent relationship between sRAGE, systolic blood pressure, and hypertension in children suggested that the AGE–RAGE axis may be altered early in life, and that sRAGE could be a compelling marker for pediatric cardiovascular risk stratification [146].

Another systematic review tried to quantitatively summarize the findings of research that investigated the relationship between circulating AGE, dietary AGE, and obesity measurements in the adult population [149]. Two meta-analyses with 11,510 participants revealed an inverse relationship between BMI and plasma AGE in healthy adults. There was no significant increase in BMI among the highest and lowest categories of dietary AGE. According to the well-known detrimental role of AGE, as well as the observation that reducing AGE intake improves adipose tissue inflammation and IR, the findings of this study were quite surprising. Although few studies were included in the analysis, the results seem to suggest that AGE plasma levels are unreliable indicators of the glycation status in obesity, maybe for different reasons. Firstly, because obesity increases the transcapillary escape rate of albumin and total body interstitial fluid volume, local protein glycation can occur at a slower rate than plasma. Secondary, adipose tissue can trap AGE differently, depending on AGE chemical properties. Lastly, the type of quantified AGE can be different associated with a specific outcome. Methylglyoxal-derived hydroimidazolone seems to be the most reliable indicator of the body’s AGE. However, until the present, it has been measured in the evaluation of AGE intake rather than the circulating pool [126,150]. Also in adolescents, CML was associated with adiposity and the risk of adiposity-related comorbidities [126,151].

**Figure 2:**
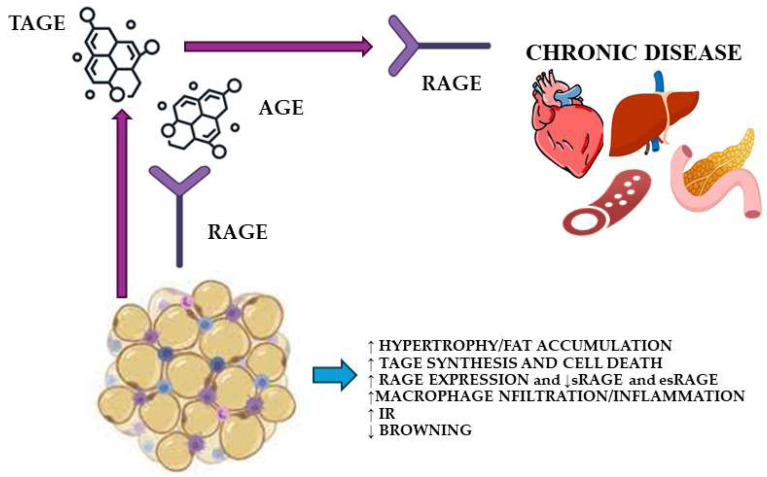
Role of sRAGE as a biomarker in obesity and obesity-related complications [4,5,53,79,80,81,86,87,108,109,110,112,114,116,117,118,119]. BMI, body mass index; VAT, visceral adipose tissue; EAT, epicardial adipose tissue; MS, metabolic syndrome; CVD, cardiovascular diseases; LDL, low-density lipoprotein cholesterol; TG, triglycerides; BP, blood pressure; HDL, high-density cholesterol; FMD, flow-mediated vasodilation; HOMA-IR, homeostatic model of IR; IGT, impaired glucose tolerant. Arrows means a mechanisms is activated or decreased.

Considering that plasma levels of sRAGE are affected by obesity, and that obesity is, in turn, a risk factor of CVD, sRAGE and AGE could be potential risk markers for CVD. Many excellent papers are focused on this topic. Some of these studies aimed to identify a potential association between sRAGE and traditional cardiovascular risk markers, which are known to be affected by overweight and obesity. In a study performed on childhood obese individuals, HDL-cholesterol levels were an independent predictor of sRAGE serum levels, suggesting that the HDL-cholesterol and sRAGE may play a critical role in lowering the cardiometabolic risk [125]. Another study including obese children confirmed the observed correlation between sRAGE levels and HDL-cholesterol [127]. IR is an additional risk factor for CVD risk. Despite the well-established role of the AGE–RAGE system in the development of IR in obesity, research on the connection between sRAGE and IR markers has been conflicting. Flores-Ramírez A. et al. [127] observed a direct association between sRAGE and HOMA-IR (homeostatic model assessment for IR) in obese children, but only in the male subgroup. He C. et al. [123] found an inverse correlation only in males. A significant correlation was found in adolescents of both genders using linear regression analysis adjusted for age and gender, but the significance was lost after further controlling for BMI. Therefore, BMI seems to be the major predictor of sRAGE levels in adolescents. sRAGE, but not esRAGE, correlated indirectly with HOMA-IR and directly with whole-body insulin sensitivity index in the work by D’Adamo et al. [128]. In obese adult women, sRAGE levels were inversely associated with HOMA-IR and other metabolic risk parameters, including central obesity, increased blood pressure, LDL-cholesterol, and triglycerides [124]. Miranda E. et al. observed that, in normal glucose tolerant individuals, sRAGE, cRAGE, and esRAGE were lower in obese than normal-weight individuals. Individuals with impaired glucose tolerance or DM (IGT-DM) had lower sRAGE, cRAGE, and cRAGE:esRAGE ratio levels than normal glucose tolerance (NGT) individuals, both in the normal weight and obese groups. Obese IGT-DM showed increased levels of cRAGE and cRAGE:esRAGE compared to NGT. A higher risk of developing DM was independently linked to lower levels of sRAGE and cRAGE, which were seen in both obesity and IGT [129]. This study confirmed that obesity and glucose tolerance can both alter sRAGE levels, but obesity seems to be the main factor reducing esRAGE. Understanding the processes underlying the modulation of sRAGE and its forms could lead to the discovery of new therapeutic options in the field of in obesity and DM. Flow-mediated vasodilation (FMD) and arterial stiffness are indicators of endothelial dysfunction and vascular structural damage that occurs in the early phase of the atherosclerotic process. sRAGE was found to be inversely correlated with FMD and directly with arterial stiffness index in adolescents with obesity [126]. Other studies found similar relationships in adults. sRAGE directly correlated with arterial stiffness and intima media thickness and inversely with FMD [130]. These studies imply potential roles for sRAGE as a counter-regulatory system against the vasotoxic effects of AGE and as a biochemical marker of compromised endothelial function. More prospective research is required to fully understand the cause-effect connection between AGE–RAGE and the established risk factors for CVD. Our group investigated the potential role of sRAGE as a CVD biomarker, too [91]. In multiple stepwise regression research aimed to highlight the importance of fat distribution in apparently healthy women of various BMI, EAT volume was the only predictor of sRAGE. Given the significance of EAT as a cardiovascular risk factor [152,153,154], and the fact that the drop in sRAGE levels occurred before the appearance of metabolic diseases, sRAGE measurement could be an early predictor of cardiometabolic risk. Another study found that, in apparently healthy obese women, central obesity was related to decreased sRAGE levels and elevated inflammatory markers, despite the presence or absence of traditional cardiometabolic risk factors [131]. The observed alterations in non-traditional risk markers, such as sRAGE, lead to the conclusion that obesity should not be called “healthy or unhealthy” only on the basis of the traditional markers. In this contest, sRAGE may be a future valuable marker for stratifying metabolic risk. Data from 2206 apparently healthy adolescents (51% girls) aged 15–19 years confirmed a decline in sRAGE and esRAGE levels before the development of metabolic syndrome. Traditional and nontraditional cardiometabolic risk markers explained only a small proportion of sRAGE and esRAGE variability (8–11%) [132]. Therefore, other still unknown mechanisms can regulate the declines in sRAGE and esRAGE levels in obesity. Figure 2 summarizes the role of sRAGE as a biomarker in obesity and obesity-related complications.

Several additional studies have been published on this topic, but they are not mentioned here since they looked at the role of sRAGE and its many variants as CVD risk markers in individuals who had been diagnosed with specific conditions, such as DM and CKD, regardless of obesity. The research stated in this section suggested that an increase in adipose tissue, particularly in the visceral area, is associated with changes in the pathways that regulate sRAGE generation, but the mechanisms have yet to be defined. We can suppose that AGE synthesis and accumulation in fat tissues activate RAGE and promote RAGE expression. Further up-regulation of RAGE, down-regulation of esRAGE secretion, and variations in the activity of RAGE-cleaving enzymes can all influence circulating sRAGE levels. More research on specific AGE is required to fully understand their significance as adiposity markers.

## 6. Potential Strategies for Reducing AGE–RAGE System Activation: From Preclinical to Clinical Studies

Potential strategies to minimize AGE–RAGE activation can include the following: (1) reducing AGE consumption, (2) reducing AGE production and accumulation, (3) increasing sRAGE circulating levels, (4) blocking circulating AGE, and (5) inhibiting RAGE. These goals could be met in part by the diet, which, in some situations, has been demonstrated to reduce AGE while boosting circulating levels of sRAGE. Table 1 summaries the results of studies which describe the beneficial effects associated with AGE–RAGE modulation. However, only a few of them evaluated whether lowering AGE and increasing sRAGE reduced obesity and obesity-related CVD risk. This opens the possibilities for new potential promising studies in this field.

AGE levels can be decreased by lowering the intake of food rich in AGE, such as red meat, animal fat, cheese, sweetened food, and foods cooked at high temperature in dry heat (frying, broiling, grilling, and roasting). Kanikowska D. et al. observed that obese people who followed a three-week diet with a 25–30% reduction in daily caloric supply (a reduction of 500–1000 kcal in relation to total energy requirement) did not show significant changes in sRAGE levels, but they did experience a decrease in EN-RAGE (extracellular newly identified receptor for advanced glycation). This cytosolic protein, also known as calgranulin C or S100A12, is released in response to cell stress and initiates a RAGE-mediated inflammatory response [155]. The authors suggested that EN-RAGE quantification could be more valuable than sRAGE in identifying the early favorable effects of caloric restriction. Another study found that restricting calories by 300–500 kcal/day for 8 weeks was unlikely to enhance lower sRAGE levels in obese people, although it could improve other traditional cardiovascular risk markers [156]. Popp C.J. et al. investigated whether baseline sRAGE isoforms predicted changes in fat mass, fat-free mass, resting energy expenditure, and adaptive thermogenesis during a three-month weight reduction period. They found moderate and variable weight loss, no significant relationship between baseline sRAGE forms and changes in fat mass and fat free mass, but a positive association with changes in resting energy expenditure that was independent of glycaemic control. Although the contribution of AGE and RAGE pathway in energy balance requires further research, this work emphasized additional involvement of RAGE in controlling energy expenditure [157]. A systematic study evaluated the effects of weight loss achieved by energy-restricted diets and bariatric surgery on serum AGE and sRAGE forms in overweight and obese individuals [158]. Energy-restricted diets for 2–6 months decreased AGE levels, possibly due to changes in dietary consumption and decreasing endogenous AGE production. The results regarding sRAGE were variable. In 6–36-month longitudinal trials following bariatric surgery, sRAGE levels increased in three studies, remained constant in two, and dropped in one. Higher baseline sRAGE levels were associated with greater weight loss. The major conclusion of this analysis is that sRAGE can be used as a biomarker to identify patients who would benefit more from an intervention program [159,160,161,162,163,164]. Changes in sRAGE concentration following bariatric surgery also correlated with changes in fasting insulin, 1 and 2 h postprandial glucose, HOMA-IR, triglycerides, and γ-glutamyl transferase, thus confirming that sRAGE modulation is associated with CVD risk in obese patients [159].

Several AGE-lowering compounds and inhibitors of AGE synthesis have been developed in the past. Some of them also progressed to clinical trials, whereas others were only evaluated in pre-clinical studies. Due to side effects or because companies have discontinued activities, some compounds have been dismissed and some studies have been stopped. A more detailed description of these compounds (Algebrium Chloride, Aminoguanidine, Benfotiamine, Carnosine, OPB-9195, Piridoxamine, and Thiamine) can be found in a previous paper from our group [51]. GLO-1, the endogenous enzyme that blocks AGE synthesis by promoting the degradation of dicarbonyl compounds, could be another interesting target for controlling AGE synthesis. GLO-1 is not available for clinical studies, but it has been shown that its levels may be up-regulated by some nutritional components, such as trans-resveratrol combined to hesperetin, and lycopene [165,166]. In addition, other plant extracts with antioxidant properties may reduce AGE synthesis and RAGE-induced oxidative stress, including date seed powder, the polysaccharide AAP-2S extracted from Auricularia auricula, and 1, 2, 3, 4, 6-Penta-O-Galloyl-dGglucose, a highly galloylated polyphenol that can be extracted by tea plants or chemically synthesized [167,168,169].

Considering the role of sRAGE as a decoy receptor, increasing its concentration could be another interesting strategy to reduce AGE-related side effects. We have previously discussed how lower sRAGE levels are associated with an increased CVD risk in obesity. Some drugs, like statins, can increase sRAGE level by inducing RAGE shedding. By performing this, they potentially prevent the development of RAGE-mediated pathogenesis [170,171,172]. Plasma levels of sRAGE were significantly increased in patients who received metformin and ACE (angiotensin-converting-enzyme) inhibitors [173]. The combination of enalapril and lercanidipine was better than the single monotherapies in reducing not only blood pressure, but also in improving sRAGE [174]. In streptozotocin-induced diabetic rats, ramipril treatment reduced the accumulation of AGE by increasing RAGE synthesis and shedding, and therefore increasing, sRAGE levels [175]. A metanalysis described that, in patients affected by chronic diseases with concomitant hypovitaminosis D, vitamin D supplementation is a useful strategy to reduce AGE-induced complications by increasing sRAGE levels [176].

The use of genetically engineered sRAGE is another intriguing approach to block AGE and reducing RAGE activation. To date, sRAGE administration has been studied only in pre-clinical models, not in humans, but with promising results. However, most of these studies were performed in clinical settings different from obesity, including subarachnoid hemorrhage, ischemia/reperfusion-induced acute kidney injury, acute respiratory distress syndrome, liver fibrosis, and myocardial ischemia/reperfusion injury [177,178,179,180,181].

Some drugs also have the potential of inhibiting RAGE expression. This group includes statins, angiotensin II receptor blockers, rosiglitazone, and the glucagon-like peptide-1 receptor agonists [182,183,184,185,186]. Interestingly, in vitro, the glucagon-like peptide 1 agonist Liraglutide reduced membrane RAGE by stimulating the shedding activity of the enzyme “Disintegrin and Metalloproteinase Domain-Containing Protein 10” [187]. Recently, Empagliflozin, a sodium-glucose co-transporter inhibitor, reduced the oxidative stress and inflammatory reactions in the adipose tissues of db/db mice due to AGE–RAGE activation [188].

Pharmacological antagonism of RAGE signaling seems to also be a promising strategy, since it promoted thermogenesis, healthy body mass and composition, and metabolism in mice [189]. No data are available about its potential at the cardiovascular level. The same conclusion could be taken by considering the potential beneficial effects of other compounds that, by blocking RAGE interactions with the formin DIAPH1, inhibit RAGE signaling once engaged by AGE [190]. Some of these compounds suppressed RAGE ligand-mediated functions, such as the migration and production of inflammatory mediators [191,192]. Amon them, the compound called “RAGE229” reduced myocardial infarction and cardiac dysfunction in diabetic mice as well as plasma concentrations of pro-inflammatory cytokines [191,192]. Future pre-clinical and clinical trials testing the effects in obesity and its complications are the long-term goal for these compounds.

Azeliragon is an oral, small-molecule, phase 2-ready compound, administered once-daily, that inhibits RAGE interactions with its natural ligands. The molecule was originally developed for Alzheimer’s disease and clinical safety data, involving over 2000 patients treated for up to 18 months, indicating a high level of safety and tolerability of the compound [193,194]. Phase 2 clinical studies are still ongoing for treatment of glioblastoma, brain metastasis, pancreatic cancer, breast cancer, and the prevention of acute kidney injury in hospitalized pneumonia patients. Preclinical studies have shown that Azeliragon has a favorable effect and safety profile in preventing metastatic triple-negative breast cancer and pancreatic cancer [195,196]. Up to now, no preclinical studies have addressed the usefulness of these compounds in the field of obesity and related CVD risks. However, in the metabolic field, azeliragon was shown to ameliorate streptozotocin-induced diabetic neuropathy and diabetes-induced alteration of vessels in the retina and kidneys [197,198,199].

**Table 1:** Potential strategies for reducing AGE–RAGE system activation. ↓: decrese; ↑, increase.

Mechanisms	Effects	Ref.
**↓ AGE INTAKE**	- Moderate CR and physical activity by decreasing inflammation reduces EN-RAGE, but not sRAGE and sRAGE - A modest weight reduction is unlikely to improve decreased sRAGE - A 3-month diet did not affect sRAGE forms, but sRAGE predicts energy expenditure - CR diets and bariatric surgery reduced serum AGE - Weight loss induced by bariatric surgery increased sRAGE	[155] [156] [157] [158] [159]
**↓ AGE**	- tRES and HESP, at concentrations achieved clinically, synergized to increase Glo1 expression - Streptozotocin-induced diabetic rats lycopene increased Glo-1 - PGG could be an effective agent to block Glu/MGO-triggered glycation in vitro - AAP-2S inhibited AGE synthesis in vitro - DSP supplementation reduced in human pentosidine levels - In mice, Liraglutide treatment reduced serum AGE levels	[165] [166] [167] [168] [169] [184]
**↑ sRAGE**	- DSP supplementation increased sRAGE in humans	[169]
	- Enalapril/lercanidipine increased sRAGE in humans	[174]
	- In diabetic rats, ACEi reduced the accumulation of AGE in DM partly by increasing the production and secretion of sRAGE into plasma	[175]
	- In animal and human, Vitamin D treatment increased sRAGE levels, particularly in vitamin D-deficient situations	[176]
**BLOCKING AGE**	- sRAGE protected against liver fibrosis - In pigs, RAGE antagonism and sRAGE decreased lung inflammation - sRAGE inhibits I/R-induced apoptosis, both in the hearts of mice and cardiomyocytes - RAGE administration prevented renal tubular damage in models of ischemia/reperfusion-induced AKI - sRAGE effectively lessened microcirculation impairment and vascular injury after SAH - Empagliflozin reduced the AGE–RAGE-oxidative stress- induced inflammatory reactions in the adipose tissues of db/db mice	[177] [178] [179] [180] [181] [188]
**INHIBITING** **RAGE EXPRESSION/** **RAGE ACTIVATION**	- PPARgamma agonists inhibit RAGE expression in vitro	[182]
	- In mice, losartan attenuated hepatic I/R-induced RAGE expression	[183]
	- In mice, liraglutide reduced the expression of RAGE in the aorta	[184]
	- In vitro, statins decreased RAGE expression	[185]
	- In mice, liraglutide downregulated kidney RAGE	[186]
	- In vitro, liraglutide reduced the number of intact RAGE on the cell surface by promoting its shedding	[187]
	- The RAGE inhibitor azeliragon had promising results on slowing the loss of cognition in preclinical AD models and in a Phase2b study	[194]
	- The RAGE inhibitor azeliragon significantly inhibited tumor growth in a pancreatic cancer xenograft model	[195]
	- The RAGE inhibitor azeliragon suppressed metastasis in triple-negative breast cancer	[196]
	- Small-molecule RAGE antagonists blocked suPAR signaling suPAR-mediated inflammatory responses in vitro podocytes	[198]
	- The RAGE inhibitor azeliragon ameliorated streptozotocin-induced diabetic neuropathy	[199]
**BLOCKING RAGE SIGNALING**	- In mice, antagonism of RAGE signaling by RAGE229 optimized healthy body mass and composition and metabolic fitness - Antagonism of RAGE signaling by small-molecule competitive inhibitors regulated signaling networks involved in inflammation and cell migration in vitro and in vivo - Targeting RAGE signaling with RAGE229 mitigated diabetic complications in rodents by attenuating inflammatory signaling	[189] [190] [192]

CR, calorie restriction; EN-RAGE, receptor for advanced glycation end product-binding protein; sRAGE, soluble receptor for advanced glycation end products; esRAGE, endogenous secretory receptor for advanced glycation end products; AGE, advanced glycation end products; tRES, trans-resveratrol; HESP, hesperetin; Glo1, glyoxalase-1; PGG, Penta-O-galloyl-β-d-glucose; Glu/MGO, glucose/methylglyoxal; AAP-2S, Auricularia auricular polysaccharide; DSP, date seed powder; ACEi, angiotensin-converting enzyme inhibitor; DM, diabetes mellitus; I/R, ischemia/reperfusion; AKI, acute kidney injury; SAH, subarachnoid hemorrhage; AD, Alzheimer Disease; suPAR, soluble urokinase plasminogen activator receptor.

## 7. Summary and Conclusions

The AGE–RAGE system is a pathway involved in adipose tissue expansion and remodeling, and it is crucial in sustaining chronic inflammation, oxidative stress, IR, and lipid metabolic changes, all of which can increase the risk of CVD. Increased AGE synthesis in adipose tissue can in turn contribute to increasing plasma AGE, which can exert RAGE-dependent and RAGE-independent negative effects in the cardiovascular system. Reduction in circulating sRAGE has been identified as an early indicator of obesity-related CVD risk. Its concentration falls before changes in the levels of other classic cardiovascular risk factors become evident. Preclinical research suggests that lowering AGE and blocking the AGE–RAGE pathway could be an effective strategy for reducing fat accumulation and CVD risk. More clinical trials are needed to determine the therapeutic potential of sRAGE modulation and RAGE inhibition in reducing obesity-related cardiovascular risks.

## Data Availability

Not applicable.
